# METTL3, an Independent Adverse Prognostic Factor for AML, Promotes the Development of AML by Modulating the PGC‐1α–MAPK Pathway and PGC‐1α–Antioxidant System Axis

**DOI:** 10.1002/cam4.70771

**Published:** 2025-04-02

**Authors:** Yuqian Tang, Xiaoyan Liu, Wu Ye, Xiaojia Wang, Xiaoyu Wei, Yiwen Du, Ying Zhang, Yuping Gong

**Affiliations:** ^1^ Department of Hematology West China Hospital, Sichuan University Chengdu Sichuan China; ^2^ Department of Hematology First Affiliated Hospital of Zhengzhou University Zhengzhou Henan China

**Keywords:** acute myeloid leukemia, m^6^A RNA methylation, METTL3, PGC‐1α, WTAP

## Abstract

**Background:**

m^6^A represents a prevalent epigenetic modification of mammalian mRNAs. Studies have demonstrated that m^6^A RNA methylation‐modifying enzymes play crucial roles in the onset and progression of AML. However, their clinical relevance remains undefined, and the mechanisms underlying their modulation of AML have yet to be elucidated.

**Results:**

The expression levels of the m^6^A RNA‐modifying enzymes METTL3, METTL14, WTAP, FTO and ALKBH5 were elevated in AML patients. METTL3‐positive AML is often accompanied by DNMT3A mutations and is also an independent poor prognostic factor for AML patients. Following METTL3 knockdown, we observed a decrease in the m^6^A level of the mitochondrial oxidative stress gene PGC‐1α in K562 and MV4‐11 cells. We analyzed the expression levels of PGC‐1α and METTL3 mRNA in 105 patients with primary AML. The expression levels of PGC‐1α and METTL3 mRNA were positively correlated. Similar to METTL3 knockdown, PGC‐1α gene knockdown resulted in increased phosphorylation of the key signaling molecules P38, c‐Jun and ERK1/2 in the MAPK signaling pathway, and decreased mRNA levels of SOD1, GPX1, catalase and UCP2 in the antioxidant system of K562 cells. Analysis of the TCGA and GSE13159 datasets, along with samples from West China Hospital, revealed that patients exhibiting high PGC‐1α expression had a poor prognosis.

**Conclusion:**

The m^6^A methylation‐modifying enzyme METTL3 is an independent prognostic factor for poor prognosis in AML patients. PGC‐1α is a downstream signaling molecule of METTL3, and METTL3 affects its expression by regulating the m^6^A level of PGC‐1α. PGC‐1α acts as an oncogene in AML by affecting the MAPK pathway and antioxidant system.

**Abbreviations**
ALKBH5: ALKN homolog 5 RNA demethylase
AML: acute myeloid leukemia
BM: bone marrow
CR‐AML: complete remission of acute myeloid leukemia
EFS: event‐free survival
HSCT: hematopoietic stem cell transplantation
m^6^A: N6‐methyladenosine
METTL14: methyltransferase‐like protein 14
METTL3: methyltransferase‐like protein 3
mRNA: messenger RNA
NCs: normal controls
OS: overall survival
PB: peripheral blood
R/R‐AML: recurrent/refractory acute myeloid leukemia
ROS: reactive oxygen species
UCPs: uncoupling proteins
WTAP: Wilms tumor 1‐associated protein

## 1 Introduction

N6‐methyladenosine (m^6^A) RNA methylation is an epigenetic modification that occurs in eukaryotes. The modification sites are located mainly in the relatively conserved domain RRACH (*R* = A/G, *H* = A/C/U) in the termination codon and 3′ UTR of mRNA [1]. m^6^A RNA methylation is involved in the posttranscriptional metabolism of mRNAs, which includes processes such as splicing, translation, stability, and degradation, thereby modulating the expression of target genes [2, 3, 4, 5, 6].

The process of m^6^A RNA methylation is regulated by enzymes that modify m^6^A RNA, including methyltransferase complexes, demethylases, and m^6^A‐specific binding proteins. Methyltransferase complexes initiate methylation, whereas demethylases initiate demethylation to achieve a dynamic and reversible balance. Additionally, m^6^A‐specific binding proteins recognize and guide methyltransferase complexes to target mRNAs. The key components of the methyltransferase complex are methyltransferase‐like protein 3 (METTL3) [7], methyltransferase‐like protein 14 (METTL14) [8], and Wilms tumor 1‐associated protein (WTAP) [9]. Demethylases include FTO [10] and ALKBH5 [11], which is the ALKN homolog 5 RNA demethylase. The m^6^A‐specific binding proteins include YTH family proteins, YTHFD1/2/3, and YTHDC1/2 [3, 12, 13, 14].

Recently, there have been reports linking m^6^A RNA‐modifying enzymes to tumorigenesis and development [15, 16, 17, 18, 19, 20, 21]. Several studies have indicated that METTL3, METTL14, and WTAP are highly expressed in patients with acute myeloid leukemia (AML) and function as oncogenes [22, 23, 24, 25]. However, it remains unclear how these enzymes are associated with patient prognosis. This study aimed to investigate the prognostic value of METTL3, METTL14, WTAP, FTO, and ALKBH5. By identifying the most promising prognostic factors, we aimed to elucidate their role in the occurrence and development of AML.

## 2 Materials and Methods

### 2.1 Materials

The primary experimental reagents used in this study are detailed in Table S1.

### 2.2 Collection and Processing of Patient Samples

A total of 174 peripheral blood (PB) and bone marrow (BM) samples were collected from patients diagnosed with AML at the Hematology Department of West China Hospital. The Biomedical Ethics Committee of West China Hospital, Sichuan University, approved this study and waived the requirement for informed consent. Among them, 112 samples were from patients with initial AML (61 from PB and 51 from BM), whereas the remaining 24 samples were from patients with relapsed/refractory AML (R/R AML) (5 from PB and 19 from BM). Additionally, 16 samples were collected from normal controls (NCs).

### 2.3 Cell Culture

The cell lines used in this study were procured from the Laboratory of Hematology, West China Hospital. K562 cells were cultured in RPMI 1640 medium supplemented with 10% FBS; MV4‐11 cells were cultured in IMDM medium supplemented with 10% FBS; and 293 T cells were cultured in DMEM medium supplemented with 10% FBS. All cell lines were maintained at 37°C in a humidified atmosphere with 5% CO_2_.

### 2.4 Quantitative Real‐Time Polymerase Chain Reaction

The mRNA levels of cells were detected using quantitative real‐time polymerase chain reaction (qRT‐PCR). Three replicates were set for each sample. All utilized primers are listed in Table S2.

### 2.5 Western Blot Analysis

Mononuclear cells were isolated from PB or BM samples using Lymphocyte Separation Medium, followed by protein extraction from these cells or AML cell lines. For protein extraction, the cells were suspended in RIPA lysis buffer on ice for 20 min. Then, the samples were centrifuged, and the supernatant was collected. The protein concentration was standardized by the BCA method according to the instructions of the BCA Protein Assay Kit. The protein loading volume was 20 μg for each sample, and the loading volume was unified to 10 μL with SDS loading buffer. Then, the samples were denatured at ≥ 95°C for 5 min. Protein detection was performed using sodium dodecyl sulfate‐polyacrylamide gel electrophoresis (SDS‐PAGE) and western blotting, with chemiluminescence detection facilitated by enhanced chemiluminescence reagents. According to the WB exposure results, samples that did not show a band were defined as negative, and samples that showed a band were defined as positive.

### 2.6 Liquid Chromatography Tandem Mass Spectrometry

mRNA of K562 shCTR, K562 shMETTL3, MV4‐11 shCTR, and MV4‐11 shMETTL3 cells was extracted using an mRNA extraction kit and then precipitated for further use. Following precipitation, the mRNA was subjected to washing with 70% ethanol, centrifugation, removal of the supernatant, and drying at room temperature until fully dry. The sample was treated with Nuclease P1 and incubated at 37°C for 4 h, followed by the addition of 3 μL of NH_4_HCO_3_ and 3 μL of alkaline phosphatase and further incubation at 37°C for 4 h. The samples were then forwarded to the State Key Laboratory at our University for mass spectrometry analysis. Quantification of m^6^A and adenosine (A) was performed by a Shimadzu ultra‐fast liquid chromatography–tandem mass spectrometry (UFLC‐MS/MS) system equipped with a triple quadrupole mass spectrometer. Sample analysis was conducted via multiple reaction monitoring, with m^6^A monitored at an m/z transition of 282.0–150.0 and A at 136.1–119.1. Quantification was achieved by comparison to standard calibration curves, and the ratio of m^6^A and A was calculated.

### 2.7 Flow Cytometry

Cells in the logarithmic growth phase were harvested, with 2 × 10^5^ cells per well seeded into a 6‐well plate. There were three replicates for each group. The appropriate concentration of the drug was then added to continue the culture.

For apoptosis analysis, cells were harvested and resuspended in flow cytometry tubes containing 100 μL of 1× binding buffer. Annexin V‐APC and 7‐AAD were sequentially added to each tube at a ratio of 2:1. The cells were incubated in the dark on ice for 15 min. Then, 300 μL of PBS was added to each tube, and the samples were analyzed by flow cytometry.

For the cell differentiation assay, cells were incubated for 72 h, then harvested and washed with PBS. The cells in the control and experimental groups were resuspended in PBS containing 0.1 μg/mL CD11b and 0.2 μg/mL CD14 antibodies, followed by incubation in the dark for 30 min and analysis by flow cytometry.

### 2.8 Co‐Immunoprecipitation (Co‐IP)

Protein extraction was described in the “Western Blotting” section. Co‐IP was subsequently conducted with a Magna RIP RNA‐Binding Protein Immunoprecipitation Kit.

### 2.9 MTT Assay

The cells in the logarithmic growth phase were harvested, and the cell density was adjusted to 1 × 10^5^ cells/mL. Ninety microliters of cell suspension and 10 μL of complete medium were added to the control wells, whereas 90 μL of cell suspension and 10 μL of various drug concentrations were added to the experimental wells. One hundred microliters of complete medium was added to the blank wells, and three replicate wells were set up. Subsequently, 200 μL of sterile PBS was added to the peripheral wells and incubated at 37°C with 5% CO_2_. After 72 h, 20 μL of 5 mg/mL MTT solution was added to each well, and the incubation was continued. After 4 h, 100 μL of MTT dissolving solution was added to each well, and the plates were incubated at 37°C overnight in a waterproof incubator. The next day, the absorbance at 570 nm was measured using a microplate reader. The cell viability was calculated as (OD of experimental well—OD of blank well)/(OD of control well—OD of blank well) × 100%.

### 2.10 Establishment of Knockdown Cell Lines

Lentiviral vectors were used to generate stable cell lines with either METTL3 or PGC‐1a knockdown. The shRNA pLent‐U6‐GFP‐puro virus of the human METTL3 gene (NM_019852) and the pLent‐U6‐GFP‐puro control virus with an inserted nonsense sequence were purchased from Shandong Vigene Biosciences Co. Ltd. The shRNA hU6‐MCS‐Ubiquitin‐EGFP‐IRES‐puro plasmid of the human PGC‐1a gene (NM_013261) and the control plasmid hU6‐MCS‐Ubiquitin‐EGFP‐IRES‐puro (GV248) with an inserted nonsense sequence TTCTCCGAACGTGTCACGT were purchased from Shanghai Genechem Co. Ltd. The knockdown sequences and plasmid map can be found in Table S3 and Figure S1, respectively. Competent cells were used to amplify plasmids, and 293 T cells were used to package the virus. Finally, the cells were infected with the virus solution carrying the target plasmid. The medium was changed after 36–48 h, and the cells were screened with puromycin (the final concentration was 1 μg/mL) at 72 h.

### 2.11 RNA‐Seq Data Analysis

We established stable K562 shCTR‐ and K562 shMETTL3‐ transfected cell lines, which were submitted to Grain Source Technology Co. Ltd. in Beijing for transcriptome sequencing. The overall process can be summarized as follows: (1) Total RNA Sample Detection: Using agarose gel electrophoresis to analyze RNA degradation and contamination; checking RNA purity with Nanodrop; accurately quantifying RNA concentration with Qubit; assessing RNA integrity with Agilent 2100. (2) Library Construction: Enriching mRNA using magnetic beads with Oligo(dT), then fragmenting the mRNA with fragmentation buffer. Using random primers to synthesize single‐stranded cDNA, adding buffer, dNTPs, and DNA polymerase I to synthesize double‐stranded cDNA, followed by purification with AMPure XP beads. The purified cDNA undergoes end repair, A‐tailing, and adapter ligation, then size selection with AMPure XP beads, and finally PCR enrichment to obtain the cDNA library. (3) Library Quality Check: performing preliminary quantification using Qubit 2.0, diluting the library to 1 ng/μL, checking insert fragment length with Agilent 2100, and after confirming expectations, accurately quantifying effective concentration using Q‐PCR (effective concentration > 2 nM). (4) Sequencing: After passing the library quality check, pooling different libraries according to effective concentration and target data volume requirements, then proceeding with Hiseq sequencing. The RNA‐seq data had been uploaded to GEO with accession number GSE282987. Subsequently, we utilized GSEA‐seq data analysis software for RNA analysis.

### 2.12 Data Analysis

Statistical analysis and graphing were performed using SPSS 25.0 and GraphPad Prism 8.0, and the data are presented as either the mean ± standard error or mean ± standard deviation. A *t*‐test was used for comparison of quantitative data, a chi‐square test was used for comparison of qualitative data, and Fisher's exact test was used when the expected frequency was less than 5. Univariate and multivariate Cox regression analyses were used to evaluate the effects of various factors on survival. Survival curves were plotted using the Kaplan–Meier method and compared using the log‐rank test. Additionally, FDR (False Discovery Rate) was utilized for multiple comparisons. A *p* value less than 0.05 was considered to indicate statistical significance. *p < 0.05; **p < 0.01; ***p < 0.001; ****p < 0.0001.

## 3 Results

### 3.1 m^6^A‐Modifying Enzymes are Highly Expressed in AML Patients

**FIGURE 1:**
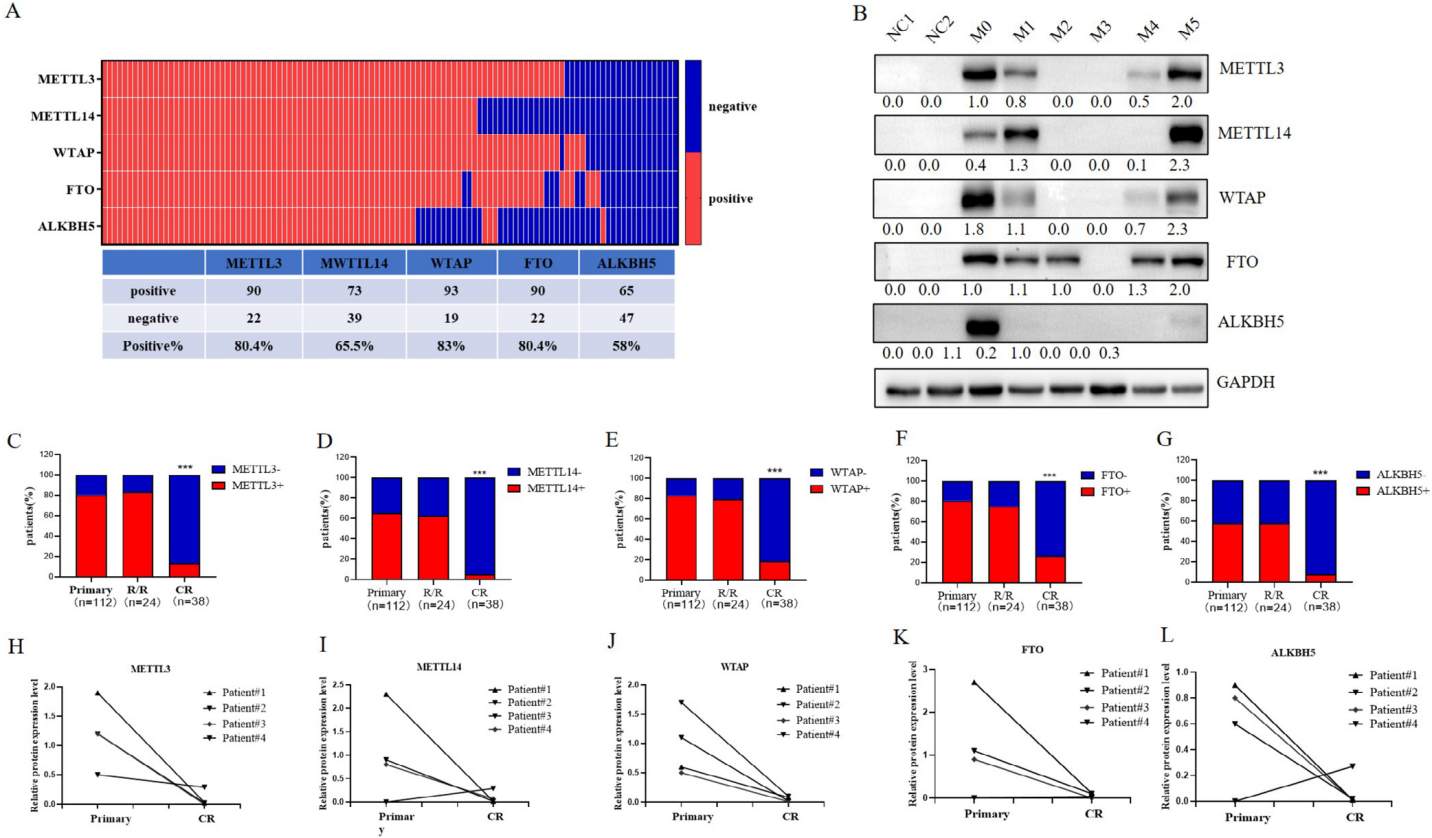
Depiction of the Expression of m^6^A RNA Modifying Enzymes in Acute Myeloid Leukemia (AML). (A) The positive rate of protein expression for m^6^A RNA modification enzymes in primary AML patients (*n* = 112); (B) A representative Western blot (WB) image demonstrating the expression of m^6^A RNA modification enzymes in normal control (NCs) and AML patients of different FAB classifications; (C–G) The expression levels of METTL3, METTL14, WTAP, FTO, and ALKBH5 at various stages of AML (*n* = 174); (H–L) The expression levels of METTL3, METTL14, WTAP, FTO, and ALKBH5 in patients at initial diagnosis and during complete remission (CR) (*n* = 4).

We first detected the protein expression levels of METTL3, METTL14, WTAP, FTO, and ALKBH5 in 112 primary AML samples and found that the percentage of positive cells positive for these 5 enzymes was 80.4% (90/112), 65.5% (73/112), 83% (93/112), 80.4% (90/112), and 58% (65/112), respectively, while no expression was detected in the NCs (*n* = 16). The difference was statistically significant (Figure 1A). A representative WB image depicting the expression of these enzymes in NCs and AML across different FAB classifications is shown in Figure 1B.

To investigate the expression levels of these 5 enzymes at different stages of AML treatment, we analyzed the overall percentage of 112 samples from newly diagnosed AML patients, 24 samples from patients with R/R‐AML, and 38 samples from patients in complete remission (CR‐AML). We found that the percentage of METTL3‐, METTL14‐, WTAP‐, FTO‐, and ALKBH5‐positive primary AML cells was not significantly different from those in R/R‐AML (*p* > 0.05), with positive rates of 83.3% (20/24), 62.5% (15/24), 79.2% (19/24), 75% (18/24), and 58.3% (14/24), respectively. However, when comparing primary AML with CR‐AML, which had positive rates of 13.2% (5/38), 5.3% (2/38), 18.4% (7/38), 26.3% (10/38), and 7.9% (3/38), the percentage of m^6^A‐modifying enzymes was significantly greater (*p* < 0.0001) in the former (Figure 1C–G). The representative WB image of Figure 1C–G was shown in Figure S2A.

We also monitored the enzyme expression levels in BM samples from 4 patients. Compared with those in the primary AML samples, the expression levels of METTL3, WTAP, and FTO in the CR‐AML samples were significantly lower (Figure 1H–L). The representative WB image of Figure 1H–L was shown in Figure S2B.

### 3.2 METTL3 and WTAP Indicate Poor Prognosis, With METTL3 as an Independent Prognostic Factor

**FIGURE 2:**
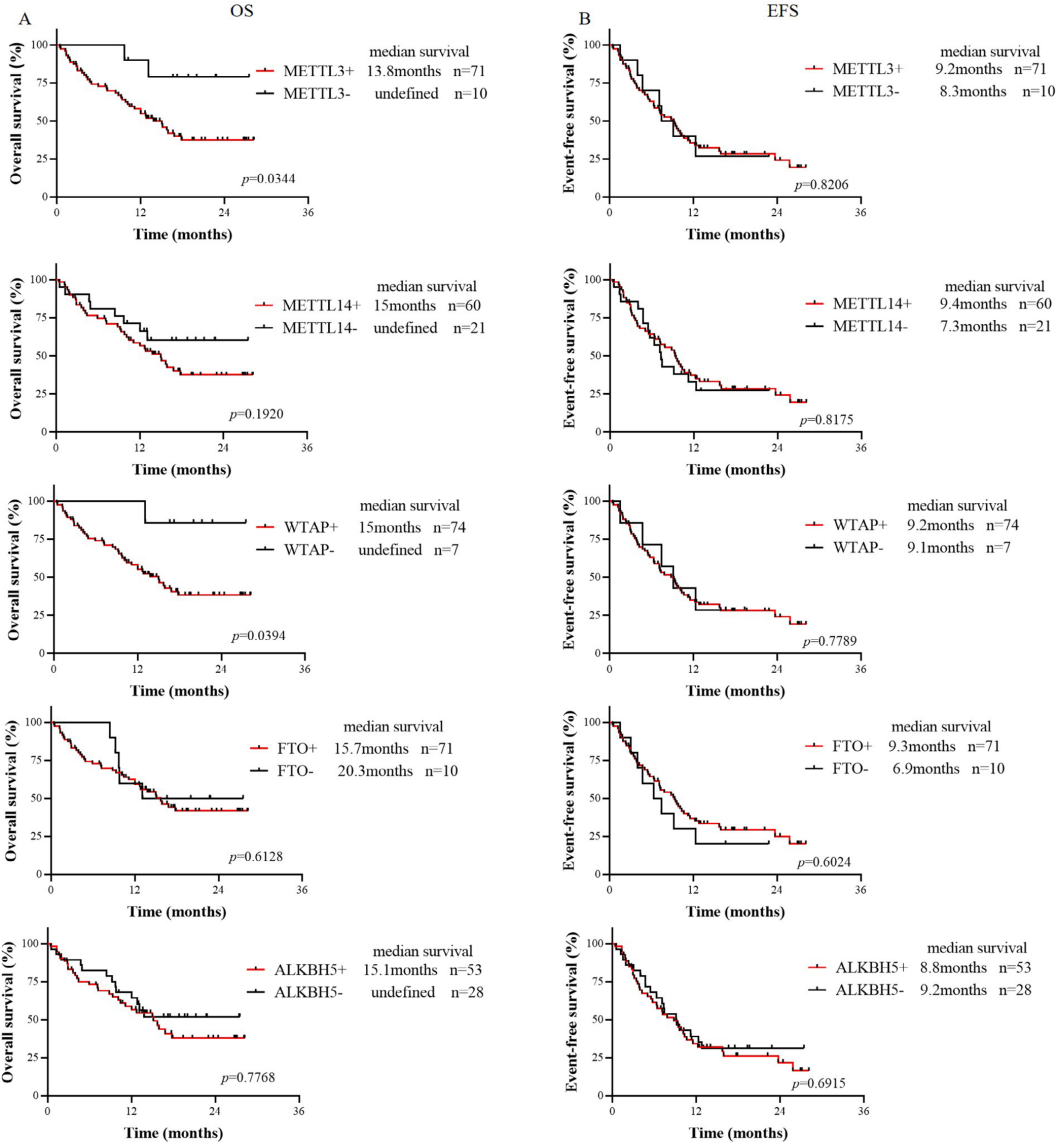
Survival Analysis of m^6^A RNA Modifying Enzymes in AML. (A) Overall Survival (OS) of patients testing positive or negative for METTL3, METTL14, WTAP, FTO, and ALKBH5 (*n* = 81); (B) Event‐Free Survival (EFS) of patients testing positive or negative for METTL3, METTL14, WTAP, FTO, and ALKBH5 (*n* = 81).

After excluding AML‐M3 patients and untreated patients, we analyzed the overall survival (OS) and event‐free survival (EFS) of 81 patients from our hospital. First, patients who were positive for METTL3, METTL14, WTAP, FTO, and ALKBH5 were defined as Group All+, patients who were negative for all of them were defined as Group All−, and the remaining patients were defined as Others. Survival analysis revealed no significant differences in OS or EFS among the three groups (Figure S3). Subsequently, the prognostic significance of the expression of the 5 modified enzymes in patients with primary AML was analyzed (Figure 2A,B). The results showed that the OS of the METTL3‐positive group was shorter than that of the METTL3‐negative group (median OS 13.8 months vs. not reached, *p* = 0.0344), and EFS was not different (*p* = 0.8206). The OS in the WTAP‐positive group was shorter than that in the WTAP‐negative group (median OS 15 months vs. not reached, *p* = 0.0394), and EFS was not different (*p* = 0.7789). There were no significant differences in OS or EFS between the METTL14‐, FTO‐, or ALKBH5‐positive group and the METTL14‐, FTO‐, and ALKBH5‐negative group. This analysis was also performed using data from the TCGA dataset, with results presented in Figure S4. We used the median as the cutoff to categorize expression levels into high and low. The results indicated that the median OS of the METTL3‐, WTAP‐positive group was longer than that of the METTL3‐, WTAP‐negative group, although this difference was not statistically significant (*p* = 0.23 and *p* = 0.20, respectively).

To determine whether these genes are independent adverse prognostic factors for AML, multivariate Cox regression analysis was performed in AML patients who were tested for AML fusion genes and 15 or more myeloid prognostic mutations (Table 1). Positive METTL3 was found to be an independent adverse prognostic factor for AML (HR: 5.635, 95% CI: 1.147–27.694, *p* = 0.033), while positive WTAP expression was not. In this study, age (HR: 2.338, 95% CI: 1.065–5.133, *p* = 0.034), TP53 mutation status (HR: 5.647, 95% CI: 1.085–29.39, *p* = 0.040), and EVI1 fusion gene status (HR: 18.35, 95% CI: 3.293–102.3, *p* = 0.001) were also found to be independent adverse prognostic factors for AML, while the presence of the FLT3‐ITD mutation and a high white blood cell count (WBC ≥ 100 × 109/L) were not.

**TABLE 1:** Univariate and multivariate analysis of OS in AML patients.

Variant	OS		
	HR	95% CI	*p*
Univariate analysis			
METTL3 (positive vs. negative)	4.025	1.723–9.401	0.0376
WTAP (positive vs. negative)	6.054	2.371–15.45	0.0413
Age (≥ 60 vs. < 60 years)	2.337	0.9143–5.974	0.0212
WBC (≥ 100 vs. < 100 × 10^9^/L)	2.353	0.8734–6.337	0.0229
FLT3‐ITD (mutant vs. wild type)	1.372	0.5728–3.288	0.4321
NPM1 (mutant vs. wild type)	0.6052	0.2506–1.461	0.3406
CEBPA (mutant vs. wild type)	0.6477	0.2632–1.594	0.4104
DNMT3A (mutant vs. wild type)	1.368	0.5437–3.441	0.4611
IDH1 (mutant vs. wild type)	2.180	0.5154–9.223	0.1334
IDH2 (mutant vs. wild type)	0.2275	0.0795–0.6504	0.1037
TET2 (mutant vs. wild type)	1.155	0.2512–5.309	0.8427
TP53 (mutant vs. wild type)	11.97	0.1124–1275	< 0.0001
KIT (mutant vs. wild type)	0.5630	0.1826–1.736	0.4236
ASXL1 (mutant vs. wild type)	1.892	0.4875–7.344	0.2228
RUNX1 (mutant vs. wild type)	0.5866	0.1234–2.788	0.5930
NRAS (mutant vs. wild type)	1.746	0.5936–5.138	0.2111
EVI1 (positive vs. negative)	11.03	0.1245–977.2	< 0.0001
CBFβ‐MYH11 (positive vs. negative)	0.8266	0.2746–2.488	0.7525
AML1‐ETO (positive vs. negative)	0.2829	0.0902–0.8872	0.1830
Multivariate analysis			
METTL3 (positive vs. negative)	5.635	1.147–27.694	0.033
WTAP (positive vs. negative)	—	—	0.202
Age (≥ 60 vs. < 60 years)	2.338	1.065–5.133	0.034
WBC (≥ 30 vs. < 30 × 10^9^/L)	—	—	0.112
FLT3‐ITD (mutant vs. wild type)	—	—	0.170
TP53 (mutant vs. wild type)	5.647	1.085–29.39	0.040
EVI1 (positive vs. negative)	18.35	3.293–102.3	0.001

**FIGURE 3:**
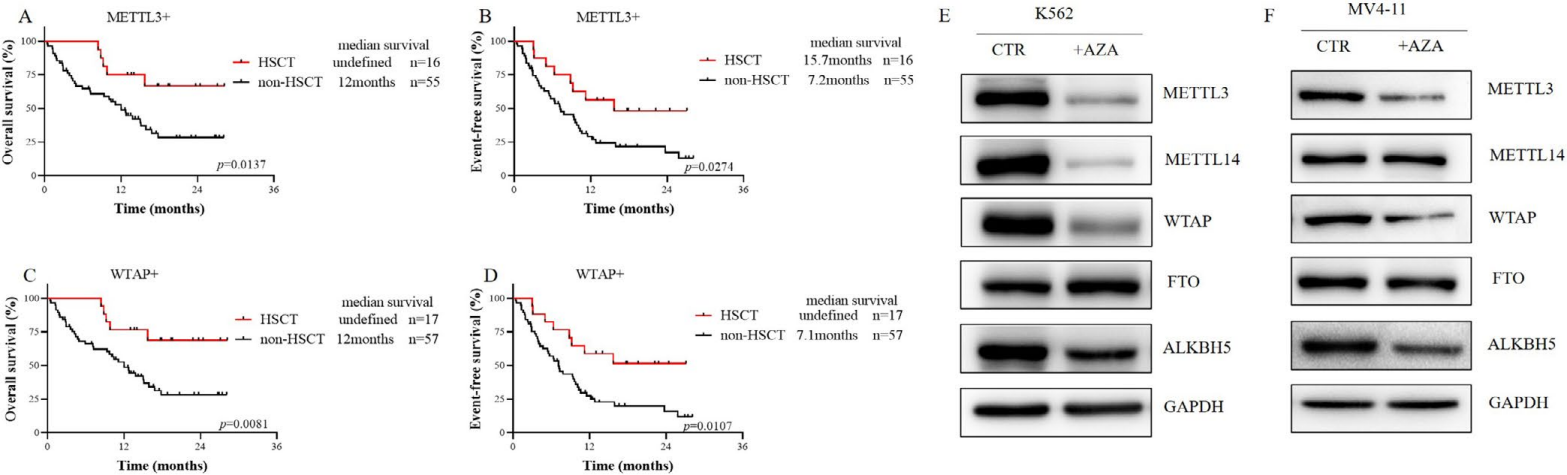
Prognostic Significance of m^6^A RNA Modifying Enzymes in AML. (A, B) Comparison of Overall Survival (OS) and Event‐Free Survival (EFS) in METTL3 positive patients who underwent Hematopoietic Stem Cell Transplantation (HSCT) and those who did not (*n* = 71); (C, D) Comparison of OS and EFS in WTAP positive patients who underwent Hematopoietic HSCT and those who did not (*n* = 74); (E, F) Effect of azacytidine on the expression of m^6^A RNA modifying enzymes in AML cells. K562 was treated with 8 μM AZA for24h, MV4‐11was treated with 5 μM 24 h.

To determine whether hematopoietic stem cell transplantation (HSCT) can improve patient prognosis, we conducted survival analyses of METTL3‐positive and WTAP‐positive AML patients who underwent HSCT and those who did not. The results revealed that for METTL3‐positive patients, the median OS was not reached in those who underwent HSCT, whereas it was 12 months in those who did not undergo HSCT (*p* = 0.0137). The EFS was 15.7 months and 7.2 months, respectively (*p* = 0.0137) (Figure 3A,B). For WTAP‐positive patients, the median OS was not reached in those who underwent HSCT, while it was 12 months in those who did not undergo HSCT (*p* = 0.0081). The median EFS was not reached vs. 7.1 months (*p* = 0.0107) (Figure 3C,D). The results indicated that the OS and EFS of patients who received HSCT were significantly longer than those of patients who did not receive HSCT.

Since METTL3 is an independent adverse prognostic factor, we next compared the clinical characteristics of METTL3‐positive and METTL3‐negative primary AML patients and found that METTL3‐positive AML patients were more likely to harbor DNMT3A mutations (*p* = 0.036) (Table S4). METTL3 has a high structural similarity to DNA methyltransferase, and azacytidine is a DNA demethylation drug that has been approved for the treatment of hematologic malignancies. Our study demonstrated that azacytidine had a lower IC_50_ in control cells than in METTL3 knockdown cells (Figure S5), and azacytidine effectively reduced METTL3 protein levels in K562 cells (Figure 3E,F). High METTL3 expression increased the sensitivity of AML cells to azacytidine.

### 3.3 METTL3 Modulates PGC‐1α Expression to Regulate the MAPK Pathway and Antioxidant System

**FIGURE 4:**
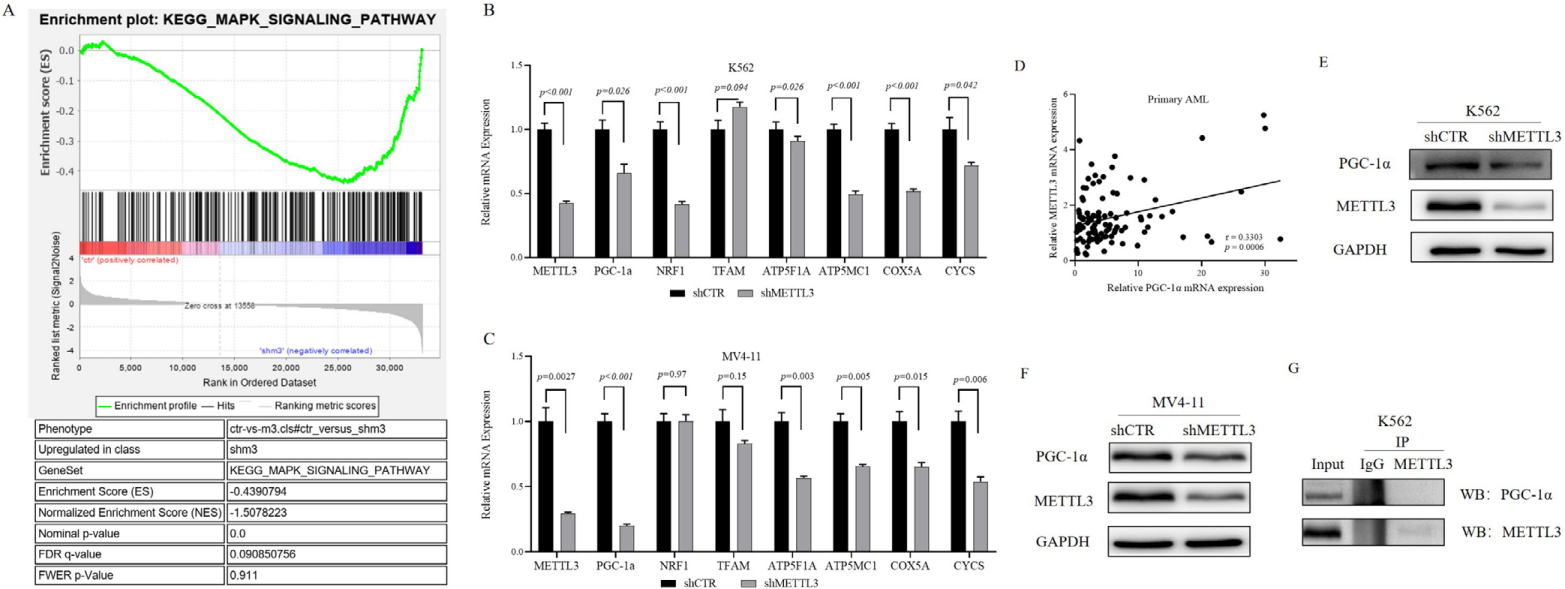
Influence of METTL3 on the Expression of the MAP Pathway and PGC‐1α. (A) GSEA analysis demonstrating the differential expression of the MAPK signaling pathway gene set in METTL3 knockdown K562 cells compared to the control group; (B, C) The impact of METTL3 knockdown on the expression of genes associated with mitochondrial biosynthesis and oxidative phosphorylation (*n* = 3); (D) Correlation analysis between METTL3 and PGC‐1α expression in primary AML cells (*n* = 105); (E, F) The effect of METTL3 knockdown on the expression level of the PGC‐1α protein; (G) Investigation of the potential interaction between the METTL3 protein and the PGC‐1α protein.

Using a lentivirus system, we successfully established K562 and MV4‐11 cell lines with METTL3 knockdown. Subsequently, we performed KEGG enrichment analysis by GSEA on the RNA‐seq data from K562 shCTR and K562 shMETTL3 cells to identify differentially expressed gene sets (|NES| > 1, NOM *p* value < 0.05, FDR *q* value < 0.25). The results suggested that genes in the MAPK signaling pathway gene set were upregulated in K562 cells with METTL3 knockdown (NES| = 1.5, NOM *p* value = 0.0, FDR *q* value = 0.091) (Figure 4A). The MAPK signaling pathway modulates cell proliferation, differentiation, and apoptosis [26]. Previous studies have reported that reactive oxygen species (ROS) can activate the MAPK pathway [27].

In mammals, mitochondria play a crucial role in regulating oxidative stress and apoptosis. To determine whether METTL3 affects genes responsible for mitochondrial oxidative stress, we detected the expression levels of genes involved in mitochondrial biosynthesis and oxidative phosphorylation in cell lines with METLL3 knockdown. As illustrated in Figure 4B,C, METTL3 knockdown significantly reduced the mRNA levels of PGC‐1α, an oxidative phosphorylation‐related gene, in K562 and MV4‐11 cells, and of NRF1 in K562 cells.

**FIGURE 5:**
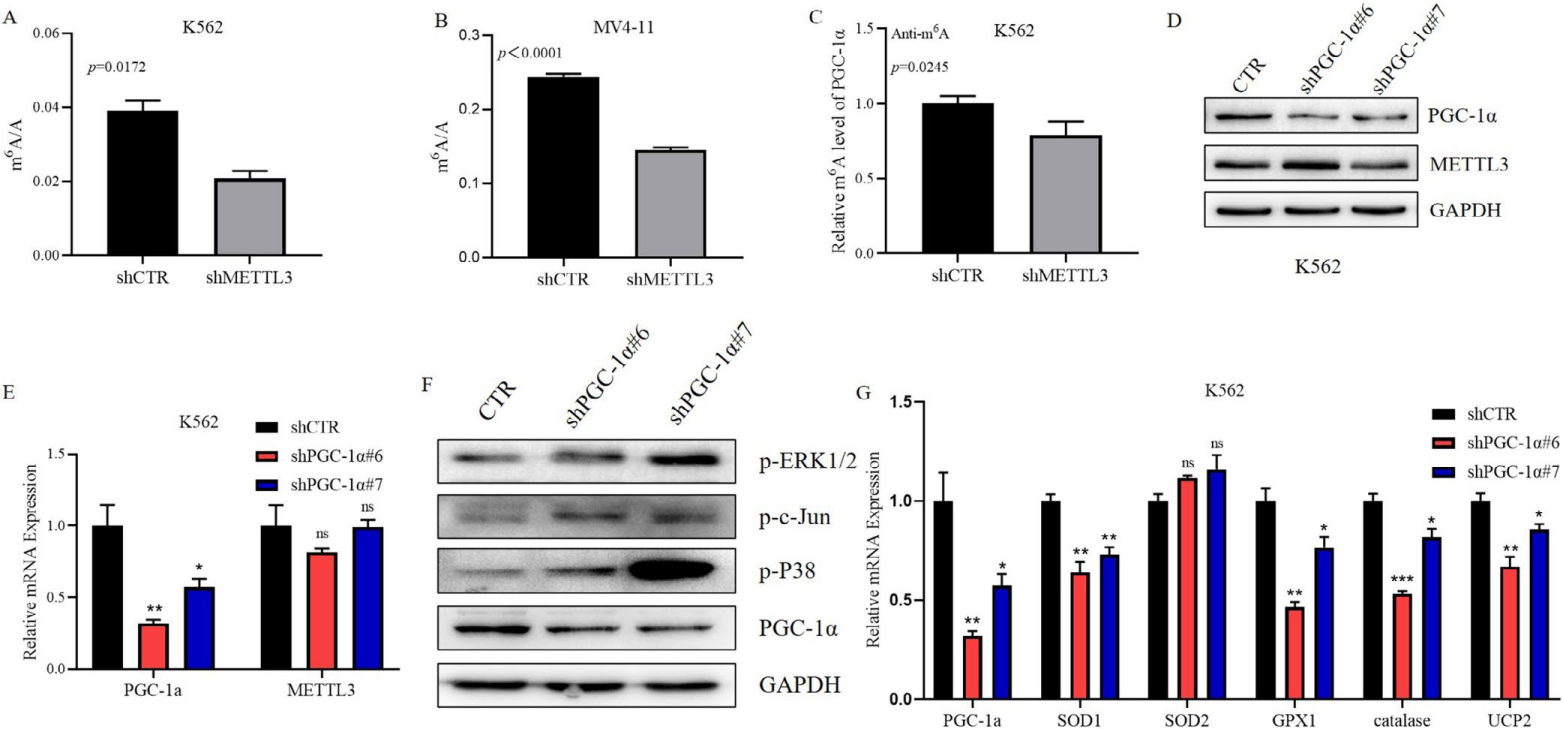
The Role of PGC‐1α in Signal Transduction Pathways. (A, B) The level of total m^6^A in AML cells following METTL3 knockdown (*n* = 3); (C) The m^6^A level of PGC‐1α in K562 following METTL3 knockdown (*n* = 3); (D) The expression level of the METTL3 protein in K562 following PGC‐1α knockdown; (E) The mRNA expression level of METTL3 in AML cells following PGC‐1α knockdown (*n* = 3); (F) The impact of PGC‐1α knockdown on the MAPK signaling pathway; (G) The effect of PGC‐1α knockdown on the mRNA levels of ROS defense system genes (*n* = 3).

Given the irreplaceable and extremely important role of PGC‐1α in modulating mitochondrial biosynthesis and oxidative phosphorylation, our study focused on its interaction with METTL3. We first analyzed the correlation between the mRNA levels of METTL3 and PGC‐1α in 105 patients with primary AML and found a positive correlation between their expression levels (*r* = 0.3303, *p* = 0.0006) (Figure 4D). Subsequently, we observed a decrease in the protein level of PGC‐1α in METTL3 knockdown K562 and MV4‐11 cells (Figure 4E,F), while the co‐IP results indicated no direct interaction between the METTL3 and PGC‐1α proteins (Figure 4G). Furthermore, we discovered a significant decrease in the total mRNA m^6^A level in METTL3 knockdown K562 and MV4‐11 cells (Figure 5A,B). This finding prompted us to test the m^6^A level of PGC‐1α mRNA, which was also found to be markedly decreased (Figure 5C). Subsequently, we constructed a PGC‐1α knockdown K562 cell line and found that the expression of METTL3 was not obviously altered at the mRNA or protein level (Figure 5D,E). Therefore, we concluded that METTL3 is the upstream gene of PGC‐1α and modulates the m^6^A level of PGC‐1α mRNA to control its expression.

To investigate whether PGC‐1α can also influence the MAPK signaling pathway, we examined alterations in key signaling molecules of this pathway in K562 cells after PGC‐1α knockdown. We found that the protein phosphorylation levels of P38, c‐Jun, and ERK1/2 in the MAPK pathway were significantly increased (Figure 5F), which indicates the activation of the MAPK signaling pathway. A literature review revealed that in addition to influencing the activity of the MAPK signaling pathway, PGC‐1α can regulate the oxidative stress state by controlling the gene expression of antioxidant systems, such as ROS detoxification enzymes and mitochondrial uncoupling proteins (UCPs), in solid tumors. To determine whether PGC‐1α can regulate the gene expression of the antioxidant system in AML cells, we detected the mRNA levels of antioxidant enzymes and UCP2 in K562 cells after knockdown of PGC‐1α. The results revealed that the mRNA levels of SOD1, GPX1, catalase, and UCP2 in K562 cells after PGC‐1α knockdown were lower than those in the control group (Figure 5G). This finding suggested a decrease in the protection of K562 cells by the antioxidant system after PGC‐1α knockdown, rendering them vulnerable to ROS.

### 3.4 Impact of PGC‐1α Knockdown on the Survival of AML Cells

**FIGURE 6:**
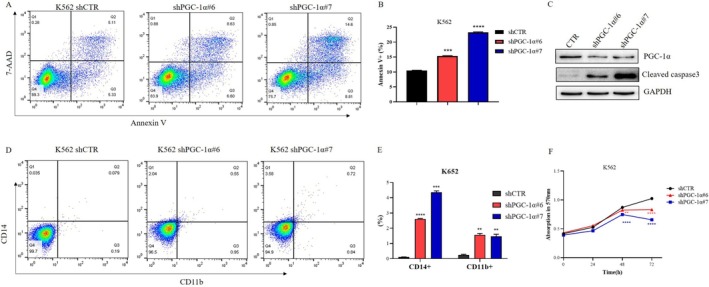
Impact of PGC‐1α Knockdown on the Survival of AML Cells. (A) Apoptosis of K562 shCTR, K562 shPGC‐1α#6, and K562 shPGC‐1α#7 cells; (B) The results from (A) are depicted in a bar chart, with the results representing the mean of 3 replicates ± standard deviation; (C) The expression of caspase‐3 in K562 shCTR, K562 shPGC‐1α#6, and K562 shPGC‐1α#7 cells was assessed using Western blot; (D) The ratio of CD14 and CD11b positive cells in K562 shCTR, K562 shPGC‐1α#6, and K562 shPGC‐1α#7 cells; (E) The results from (D) are illustrated in a bar chart, with the results representing the mean ± standard deviation of 3 replicates; (F) The impact of PGC‐1α knockdown on the proliferation of AML cells. vs. CTR: ‘ns’ indicates *p* > 0.05. vs. CTR: ‘**’ is *p* < 0.01, ‘***’ is *p* < 0.001, ‘****’ is *p* < 0.0001.

Flow cytometry was used to detect the apoptosis rate of cells in the control group and in the group after knockdown of the PGC‐1α gene; the results indicated that cells with PGC‐1α knockdown had a greater apoptosis rate (Figure 6A,B). Analysis of caspase‐3 also revealed that cells with PGC‐1α knockdown expressed more caspase‐3 (Figure 6C), suggesting that knocking down PGC‐1α leads to cell apoptosis.

We also evaluated the percentage of CD14 + CD11b + cells among both control and PGC‐1α knockdown cells. After knocking down PGC‐1α, the subset of CD14 + CD11b + cells increased (Figure 6D,E). It was suggested that the knockdown of PGC‐1α promoted the differentiation of K562 cells into mononuclear and myeloid cells.

Subsequently, an MTT assay was performed to determine the proliferation of PGC‐1α knockdown cells; the proliferation of these cells was significantly slower than that of control cells (Figure 6F).

Additionally, the cell cycle distribution of control and PGC‐1α knockdown K562 cells was analyzed, and there was no significant difference between them (Figure S6).

### 3.5 PGC‐1α Is Highly Expressed in AML Patients and Is Also an Adverse Factor for Prognosis

**FIGURE 7:**
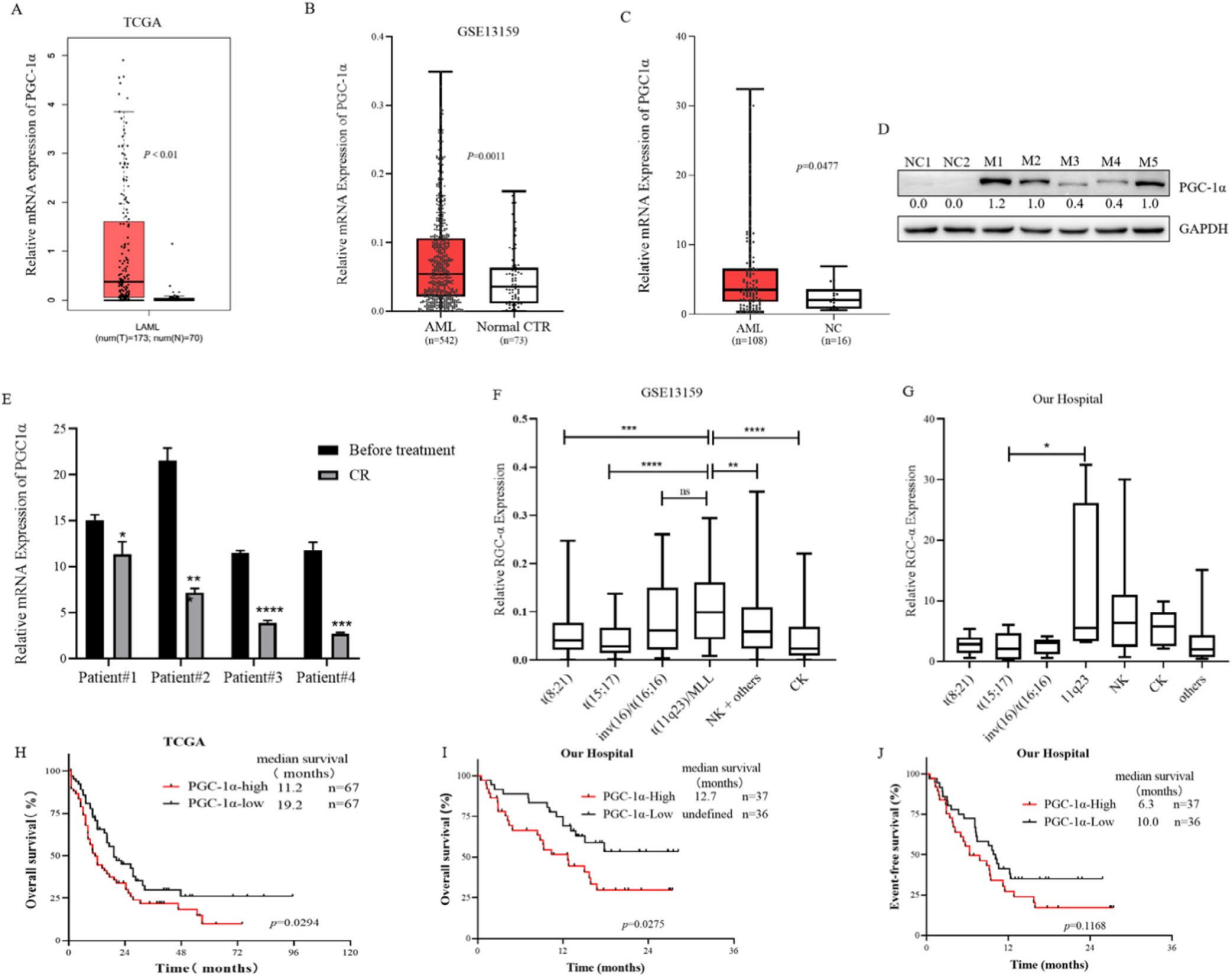
Clinical Significance of PGC‐1α. (A) Expression of PGC‐1α in the TCGA dataset for NCs (*n* = 70) and AML patients (*n* = 173); (B) Expression of PGC‐1α in the GSE13159 dataset for NCs (*n* = 73) and AML patients (*n* = 542); (C) Expression of PGC‐1α in NCs (*n* = 16) and newly diagnosed AML patients from West China Hospital (*n* = 108); (D) Representative Western Blot analysis showing PGC‐1α protein expression; (E) Expression of PGC‐1α in 4 patients at the initial diagnosis and at complete remission (CR), the results representing the mean ± standard deviation of 3 replicates; (F) Correlation between PGC‐1α expression and karyotype in the GSE13159 dataset (*n* = 40 for t(8;21); *n* = 37 for t(15;17), *n* = 28 for inv(16)/t(16;16); *n* = 38 for t(11q23)/MLL; *n* = 352 for NK + others; *n* = 47 for CK); (G) Correlation between PGC‐1α expression and chromosomal karyotype in AML patients from West China Hospital (*n* = 7 for t(8;21); *n* = 14 for t(15;17), *n* = 8 for inv(16)/t(16;16); *n* = 4 for t(11q23); *n* = 37 for NK; *n* = 5 for CK, *n* = 11 for others); (H) Overall survival (OS) in AML patients with high and low PGC‐1α expression in the TCGA database (*n* = 134); (I, J) OS and event‐free survival (EFS) in newly treated AML patients in the high and low PGC‐1α expression groups from West China Hospital (*n* = 76).

After analyzing the TCGA dataset (*p* < 0.01) (https://www.cbioportal.org), which included 173 adult AML patients and 70 NCs (Figure 7A), and the GSE13159 dataset (*p* = 0.0011) [22], which contained 542 adult AML patients and 73 NCs (Figure 7B), AML patients had higher PGC‐1α expression than NCs. We also examined the mRNA level of PGC‐1α in 108 primary AML samples from our hospital, and these samples also exhibited increased PGC‐1α expression (Figure 7C). The cut‐off used here for high or low expression of PGC‐1α was the median. Additionally, we detected PGC‐1α protein expression in 2 NCs and 5 patients diagnosed with 5 different FAB subtypes. The results revealed that PGC‐1α was barely expressed in the NCs and that 5 AMLs were expressed (Figure 7D). We also detected METTL3 and PGC‐1α expression in LSCs based on the GSE76009 dataset (Figure S7). The mRNA levels of PGC‐1α in 4 patients at primary and CR showed that PGC‐1α expression decreased when patients achieved CR (Figure 7E).

To determine the PGC‐1α expression landscape in patients with different chromosome karyotypes, we analyzed the GSE13159 dataset. PGC‐1α expression was greater in patients with 11q23, but lower in patients with t(8;21) and t(15;17), which suggests a favorable prognosis (Figure 7F). The analysis of data from West China Hospital also indicated that the expression of PGC‐1α was greater in AML patients with the 11q23 karyotype, normal karyotype, and complex karyotype, and relatively low in those with the t(15;17) karyotype (Figure 7G).

Finally, we analyzed the survival of 134 patients with AML (non‐M3 type) in the TCGA cohort. The OS of patients with AML in the group with high PGC‐1α expression was shorter than that in the group with low PGC‐1α expression (median OS 11.2 months vs. 19.2 months, *p* = 0.0294) (Figure 7H). Survival analysis of 73 patients with AML (non‐M3 type) at West China Hospital also revealed that the median OS of patients with AML with high PGC‐1α expression was shorter than that of patients with low PGC‐1α expression (median OS 12.7 months vs. not reached, *p* = 0.0275) (Figure 7I), and there was no significant difference in EFS (median EFS 6.3 months vs. 10.0 months, *p* = 0.1168) (Figure 7J). These results suggest that the high expression of PGC‐1α in AML indicates a poor prognosis.

## 4 Discussion

Our study is organized into three parts:

In the first section, we investigated the expression levels of m^6^A RNA‐modifying enzymes in AML and their prognostic value. It is generally believed that m^6^A RNA methyltransferases and demethylases play opposite roles in tumor development, so their expression levels should also be opposite. However, the methyltransferase complex (METTL3‐METTL14‐WTAP) and demethylases (FTO and ALKBH5) can both promote the proliferation of AML cells and maintain the undifferentiated state of AML cells [22, 23, 28, 29]. This is not an isolated case; DNA methyltransferase DNMT3A and demethylase TET2 are both tumor suppressor genes in myeloid tumors, and their mutations are closely related to the occurrence of myeloid tumors [30, 31]. They collaborate to inhibit lineage differentiation of HSCs [32]. In this study, the analysis of AML patient samples revealed that m^6^A RNA methyltransferases (METTL3, METTL4 and WTAP) and demethylases (FTO and ALKBH5) had significantly greater positive ratios in AML patients than in NCs (Figure 1A,B). Therefore, we speculated that the synergistic effect of m^6^A methyltransferase and demethylase promotes the occurrence and development of AML and maintains the level of m^6^A in AML cells within the range conducive to cell survival.

We observed no significant difference in the percentage of cells positive for the methyltransferases METTL3 and METTL14 or WTAP, or the demethylases FTO and ALKBH5 between primary AML samples and R/R‐AML samples, but this percentage was significantly greater than that in CR‐AML samples (Figure 1C–G), suggesting that m^6^A RNA modification enzymes may have prognostic value. Currently, there are limited reports on the relationship between m^6^A modifying enzymes and AML survival, and only a few studies have reported the poor prognosis of high expression of WTAP [25] and ALKBH5 [29, 33]. Analysis of data from the TCGA database revealed that METTL3, METTL14, and FTO were highly expressed in AML but had no prognostic significance (Figure S4) [29]. However, in the dataset obtained from our hospital, METTL3 and WTAP were poor prognostic factors for AML (Figure 2A,B), and multivariate Cox regression analysis showed that METTL3 was an independent prognostic factor (HR: 5.635, 95% CI: 1.147–27.694; *p* = 0.033) (Table 1). Additionally, we observed that HSCT prolonged the survival of METTL3‐positive AML patients (median OS not reached vs. 12 months, *p* = 0.0137; median EFS 15.7 vs7.2 months, *p* = 0.0137) (Figure 3A). FLT3‐ITD mutation was not an independent poor prognostic factor in the AML population in this study. It is speculated that most patients with FLT3‐ITD mutations are treated with drugs such as sorafenib, which prolongs the survival time. Regarding the effect of a high white blood cell count on prognosis, it may be that some patients were treated with leukocyte‐reducing drugs (such as hydroxyurea) before admission, and we did not ascertain their initial white blood cell count. As a result, they were not independent poor prognostic factors in our study.

Our study further revealed that METTL3‐positive AML patients had increased WBC counts at initial diagnosis and often had DNMT3A mutations (Table S4), suggesting that there are some correlations between m^6^A and DNA methylation. Furthermore, studies have shown that METTL3 and DNA methyltransferase are highly similar [34]; thus, we speculated that DNA demethylating drugs may be effective for METTL3‐positive AML patients, and trials have been performed to confirm this finding. The observation that the METTL3 expression level in LSCs was greater than that in non‐LSCs (Figure S7) also indicates that METTL3 inhibitors are a very promising targeted drug. To date, a small molecule inhibitor targeting METTL3 has been documented to exhibit remarkable anti‐tumor efficacy, particularly in AML [35, 36, 37, 38, 39, 40]. Nonetheless, the current approach to treating AML continues to rely on conventional chemotherapy. Although standard treatment protocols can yield complete remission in 60% of patients under 60 years old and 40%–60% of elderly patients, the recurrence rates remain elevated, and post‐recurrence survival durations remain limited. Therefore, exploring the mechanism by which METTL3 regulates AML and possible targets for treatment is highly important.

In the second section, we explored how METTL3 functions in AML. KEGG enrichment analysis of K562 shCTR and K562 shMETTL3 cells via GSEA of RNA‐seq data revealed differential expression of MAPK signaling pathway molecules between the two groups (Figure 4A). The MAPK signaling pathway connects extracellular signals with cell proliferation, differentiation, migration, and apoptosis. There are four main MAPKs in mammals: ERK1/2, JNK, P38, and ERK5, which are activated by specific MAPKKs. The activation of ERK1/2, JNK, and P38 is related to stress and participates in the regulation of cell proliferation, differentiation, and apoptosis [26]. When mitochondria produce ATP, they also produce ROS, and the ROS level in tumor cells is maintained at an appropriate level that is higher than that in normal cells but conducive to their survival. Excessively high levels of ROS can activate the MAPK signaling pathway and lead to oxidative stress and apoptosis in tumor cells [27, 41]. We discovered that METTL3 can regulate the expression of PGC‐1α (Figure 4B–G, Figure 5A–E), a gene related to mitochondrial biosynthesis and oxidative phosphorylation. It has been documented that METTL3 can collaborate with YTHDF2 to modify PGC‐1α mRNA, leading to mRNA degradation and subsequently amplifying the inflammatory response [42]. In our investigation, we similarly observed that METTL3 acts as the upstream regulator of PGC‐1α; however, intriguingly, in the context of AML, the expression levels of PGC‐1α are elevated. We found that the knockdown of PGC‐1α can also activate the MAPK signaling pathway (Figure 5F). PGC‐1α regulates cell proliferation and apoptosis by regulating the levels of antioxidant enzymes in a variety of tumors [43, 44, 45, 46]. We discovered that the mRNA levels of the ROS detoxifying enzymes SOD1, SOD2, GPX1, catalase, and UCP2 decreased in the cells with knockdown of PGC‐1α (Figure 5G). It was hypothesized that the expression of PGC‐1α decreased after METTL3 knockdown, which further decreased the expression of genes involved in the antioxidant system, thus activating the MAPK signaling pathway. We demonstrated that METTL3 influences its expression by regulating the level of PGC‐1α mRNA m^6^A, regulating the expression of genes involved in the ROS detoxification system, and negatively regulating the MAPK signaling pathway. Therefore, we propose that the METTL3/PGC‐1α/MAPK axis plays an important role in AML.

Finally, we examined the clinical significance of PGC‐1α. PGC‐1α was first reported in 1998 as a transcriptional costimulatory factor in brain brown adipose tissue and a nuclear protein of 91 kDa [47]. A significant function of PGC‐1α is to regulate mitochondrial biosynthesis and oxidative phosphorylation, which play an irreplaceable role in regulating mitochondrial biosynthesis [48]. PGC‐1α can also regulate the metabolism of tumor cells [49] and has prognostic significance in many kinds of tumors [50, 51, 52, 53, 54]. The deletion of PGC‐1α can lead to an increase in ROS levels and apoptosis in multiple myeloma after chemotherapy [55]. PGC‐1α also shapes the tumor microenvironment by regulating angiogenesis, such as by promoting angiogenesis in multiple myeloma and breast cancer [56, 57], but the underlying mechanism is still unclear. Some studies have shown that the expression of PGC‐1α in bone marrow mesenchymal cells can promote the development of AML, and high expression of PGC‐1α can reduce the ROS level in AML cells [58]. However, the mechanism of PGC‐1α in AML is still unclear.

We investigated the effects of PGC‐1α on AML cells. The findings indicated that after knocking down PGC‐1α, the proliferation of K562 cells decreased, apoptosis increased, and differentiation into monocytes and myeloid lines increased (Figure 6A–F), suggesting that PGC‐1α may act as an oncogene in AML and that these phenotypes are similar to the effects of METTL3 knockdown on AML cells [22, 59]. METTL3 is highly expressed in LSCs, but there is no difference in the expression of PGC‐1α between LSCs and non‐LSCs (Figure S7), which further confirms our conjecture that PGC‐1α is a downstream gene of METTL3.

Subsequently, we analyzed the information of newly diagnosed AML patients in the TCGA dataset, GEO database, and West China Hospital and found that PGC‐1α expression in newly diagnosed adults was significantly greater than that in NCs (*p* < 0.05) (Figure 7A–D) and decreased when AML patients reached CR (Figure 7E). Analysis of the TCGA database and survival data of patients with AML at West China Hospital revealed that the OS of patients with high PGC‐1α expression in AML was shorter than that of patients with low PGC‐1α expression (*p* < 0.05) (Figure 7H–J), suggesting that PGC‐1α is highly expressed in newly diagnosed adult AML and indicates a poor prognosis.

In summary, this study verified that METTL3 and WTAP are factors indicating poor prognosis in patients with AML; METTL3 is an independent prognostic factor, and receiving HSCT can improve the prognosis of patients with positive METTL3 and WTAP expression. METTL3 predominantly influences the survival of AML cells by regulating the MAPK pathway. PGC‐1α is a downstream molecule of METTL3, and METTL3 regulates its expression by regulating the m^6^A level of PGC‐1α mRNA. PGC‐1α can modulate the MAPK pathway and ROS detoxification system to impact the survival of AML cells and act as an oncogene, which is also a poor prognostic factor in patients with AML.

## Author Contributions

**Yuqian Tang:** conceptualization (equal), data curation (equal), formal analysis (equal), methodology (equal), validation (equal), writing – original draft (equal). **Xiaoyan Liu:** conceptualization (equal), data curation (equal), formal analysis (equal), methodology (equal), validation (equal), writing – review and editing (equal). **Wu Ye:** data curation (supporting), formal analysis (supporting), methodology (supporting), validation (equal), writing – review and editing (equal). **Xiaojia Wang:** formal analysis (supporting), methodology (supporting), validation (supporting), writing – review and editing (supporting). **Xiaoyu Wei:** data curation (supporting), formal analysis (supporting), methodology (supporting), validation (equal), writing – review and editing (equal). **Yiwen Du:** formal analysis (supporting), methodology (supporting), validation (equal), writing – review and editing (equal). **Ying Zhang:** data curation (supporting), methodology (supporting), visualization (equal), writing – review and editing (equal). **Yuping Gong:** conceptualization (lead), funding acquisition (lead), resources (lead), supervision (lead), writing – review and editing (lead).

## Ethics Statement

This study was performed in accordance with the Declaration of Helsinki. Ethical approval for this study was granted by the Biomedical Ethics Committee of West China Hospital, Sichuan University, which also waived the requirement for informed consent.

## Conflicts of Interest

The authors declare no conflicts of interest.

## Supporting information


Data S1.

## Data Availability

The data that support the findings of this study are openly available in the TCGA at https://www.cbioportal.org and in the GEO at https://www.ncbi.nlm.nih.gov/geo/query/acc.cgi?acc=GSE13159 reference number GSE13159 [22]. The data that support the findings of this study from patient samples are available from the corresponding author upon reasonable request.
