# Supplementary material for: METTL3, an Independent Adverse Prognostic Factor for AML, Promotes the Development of AML by Modulating the PGC‐1α–MAPK Pathway and PGC‐1α–Antioxidant System Axis
Source: Cancer Med. 2025 Apr 2;14(7):e70771. doi: 10.1002/cam4.70771 (PMC11962650; doi:10.1002/cam4.70771)
Supplement: Supplementary file 1 — Data S1. [file CAM4-14-e70771-s001.docx]

**Supplementary Information**

**METTL3, an independent adverse prognostic factor for AML, promotes the development of AML by modulating the PGC-1α – MAPK pathway and PGC-1α – antioxidant system axis.**

1. **Supplementary methods and materials**

| **Supplement Table 1** Main reagents and suppliers | |
| --- | --- |
| Reagents | Suppliers |
| RPMI 1640 medium | Corning |
| IMDM medium | HyClone |
| DMEM medium | Corning |
| Fetal Bovine Serum | GEMINI |
| Lymphocyte Separation Medium | TBD |
| RIPA Lysis Buffer (medium) | Beyotime Biotechnology |
| BCA Protein Assay Kit^*^ | ComWin Biotech |
| 5×SDS loading buffer | Solarbio |
| Prestained protein marker | Page ruler (26616) |
| PVDF membrane | Millipore |
| Rabbit Anti-METTL3 antibody | CST (86132) |
| Rabbit Anti-METTL14 antibody | Sigma (HPA038002) |
| Rabbit Anti-WTAP antibody | Abcam (ab195380) |
| Rabbit Anti-FTO antibody | HuaBio（ET1705-89） |
| Rabbit Anti-ALKBH5 antibody | HuaBio（ER65894） |
| Mouse Anti-GAPDH antibody | ZSGB-Bio |
| PrimeScript™ RT Master Mix | Takara（RR036A） |
| 2x SYBR Green qPCR Master Mix | Bimake（B21203） |
| GenElute™ mRNA Miniprep Kit^*^ | Sigma-Aldrich (MRN70) |
| DH5α Competent cells | TIANGEN BIOTECH（CB101） |
| Endo-free Plasmid Midi kit^*^ | Omega(D6915-03) |
| polybrene | sigma |
| Puromycin | Cayman Chemical |
| MTT | Solarbio |
| Annexin V-APC | US Everbright(Y-6026) |
| PI | US Everbright(Y-6026) |
| 7AAD | US Everbright (A6030) |
| APC anti-human CD11b | BioLgend |
| PE/Cyanine7 anti-human CD14 | BioLgend |
| METTL3 Rabbit mAb | CST (86132) |
| PGC1αMouse mAb | Proteintech (66369-1) |
| GAPDH Mouse mAb | ZSGB-Bio |
| Cleaved caspase-3 Rabbit mAb | abcam (ab195352) |
| Phospho-p38 MAPK (Thr180/Tyr182) Rabbit mAb | CST（4511） |
| Phospho-c-Jun (Ser63) Rabbit mAb | CST（2361） |
| Phospho-p44/42 MAPK (Erk1/2) Rabbit mAb | CST（4246） |
| HRP Conjugated Goat anti-Mouse IgG Goat Polyclonal Antibody | ZSGB-Bio |
| HRP Conjugated Goat anti-Rabbit IgG Goat Polyclonal Antibody | ZSGB-Bio |
| protein A/G plus-Agarose IP reagent | Santa（#J0319） |
| Magna RIP RNA-Binding Protein Immunoprecipitation Kit^*^ | Millipore (17-700) |
| Anti-N6-methyladenosine (m^6^A) | Millipore (MABE1006) |

^*^: All the kits were used according to the instructions.

| **Supplement Table 2** The primer sequences of genes in qPCR | | |
| --- | --- | --- |
| Genes | Forward primer | Reverse primer |
| GAPDH | AATTCCATGGCACCGTCAAG | TGGACTCCACGACGTACTCA |
| METTL3 | CTTCAGCAGTTCCTGAATTAGC | ATGTTAAGGCCAGATCAGAGAG |
| METTL14 | ACGGGGACTTCATTCATGCT | GAGCCAGCCTGGTCGAATTG |
| WTAP | GGATTTCACAGGGAGGGCAA | ACCCCGCACTGAGTTGATTT |
| FTO | CTCCCCGTGGAACAAAGGAA | AGCAGGTAATGTTCGGGCAA |
| ALKBH5 | GCCGTCATCAACGACTACCA | CGACACCCGAATAGGCTTGA |
| PGC-1α | AGGAAAGGAAGACCAAGCGG | AAATCTGCCCCTGCCAATCA |
| NRF1 | GCTGATGAAGACTCGCCTTCT | TACATGAGGCCGTTTCCGTTT |
| TFAM | CGCTCCCCCTTCAGTTTTGT | CCAACGCTGGGCAATTCTTC |
| ATP5F1A | AAACTGGAGCCCAGCAAGAT | CCATCAGCCCTGATAGTGCC |
| ATP5MC1 | GAACCGTGTTTGGCAGCTTG | GACAGGGCAAAGCCAAGAATG |
| COX5A | TGGCTATCCAGTCAGTTCGC | TGTTACCCAGCGAGCATCAA |
| CYCS | ATTATGAAGTGTTCCCAGTGCC | GCTGTGTAAGAGTATCCAGGGG |
| SOD1 | GACCAGTGAAGGTGTGGGGA | AAGTCTCCAACATGCCTCTCTT |
| SOD2 | GCTGGAAGCCATCAAACGTG | GCAGTGGAATAAGGCCTGTTG |
| GPx1 | CCGGGACTACACCCAGATGA | CTTGGCGTTCTCCTGATGCC |
| catalase | TCATCCAGAAGAAAGCGGTCA | AGATCCGGACTGCACAAAGG |
| UCP2 | GTCCGGTTACAGATCCAAGG | CATTGTAGAGGCTTCGGGGG |
| Mito | CACTTTCCACACAGACATCA | TGGTTAGGCTGGTGTTAGGG |
| B2M | TGTTCCTGCTGGGTAGCTCT | CCTCCATGATGCTGCTTACA |

| **Supplement Table 3** Gene shRNA sequences | | | | | |
| --- | --- | --- | --- | --- | --- |
| Gene | 5’ | stem | loop | stem | 3’ |
| METTL3 | CCGG | GCCAAGGAACAATCCATTGTT | CTCG  AG | AACAATGGATTGTTCCTTGGC | TTTTTG |
| PGC-1α#6 | CCGG | GTGCTACCTGAGAGAGACTTT | CTCGAG | AAAGTCTCTCTCAGGTAGCAC | TTTTTG |
| PGC-1α#7 | CCGG | AGGCTGAAGAGGGAAGAATAT | CTCGAG | ATATTCTTCCCTCTTCAGCCT | TTTTTG |

| 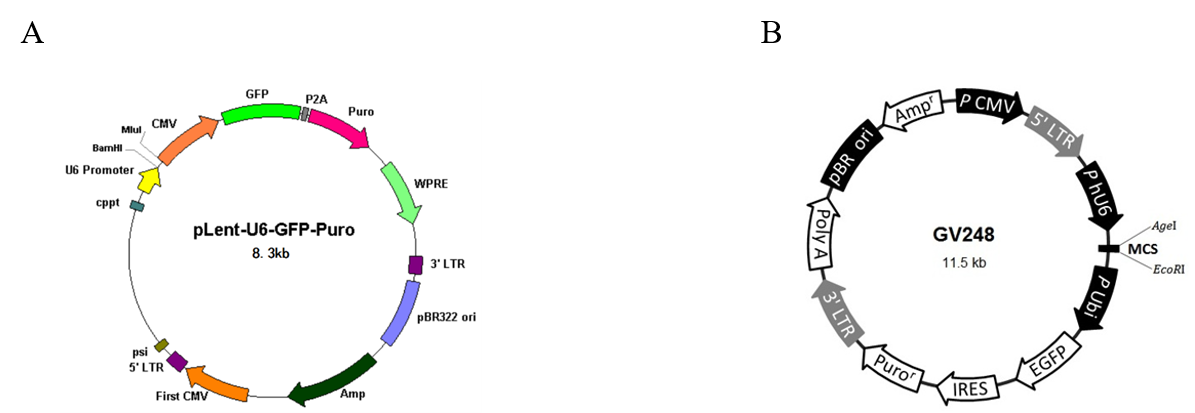 |
| --- |
| **Supplement figure 1** A. pLent-U6-GFP-Puro plasmid profile. B. hU6-MCS-Ubiquitin-EGFP-IRES-puro plasmid profile. |

1. **Supplementary figures and tables**

| **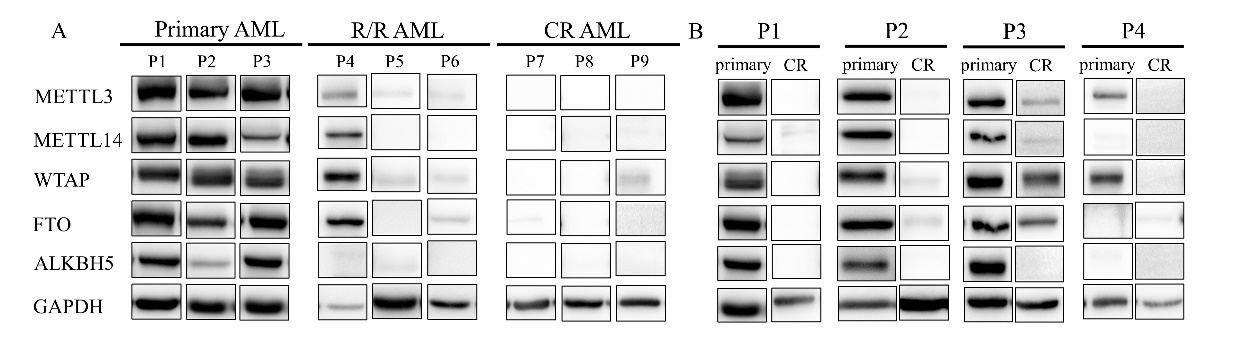** |
| --- |
| **Supplement figure 2** (A) A representative WB image demonstrating the expression of m^6^A RNA modification enzymes in AML patients at various stages of AML; (B) A representative WB image demonstrating the expression of m^6^A RNA modification enzymes in AML patients at initial diagnosis and during CR. |

| **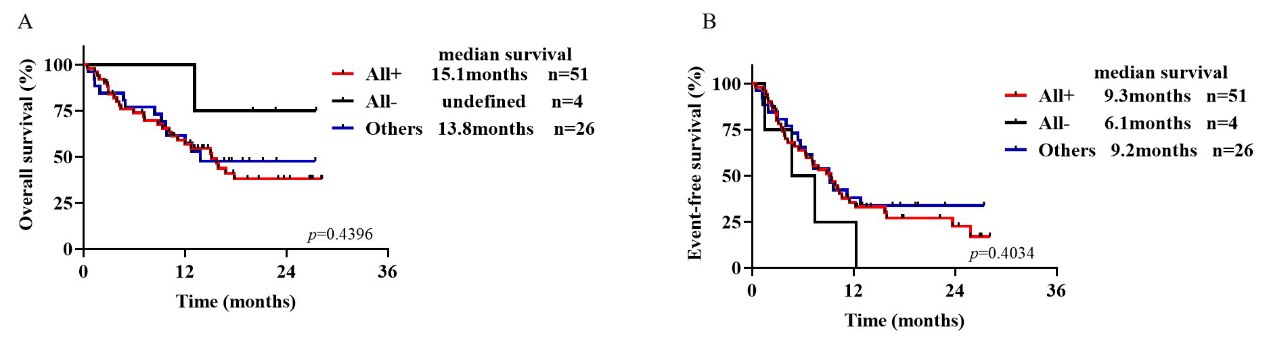** |
| --- |
| **Supplement figure 3** OS and EFS for patients in Group All+, Group All- and Others. |

| **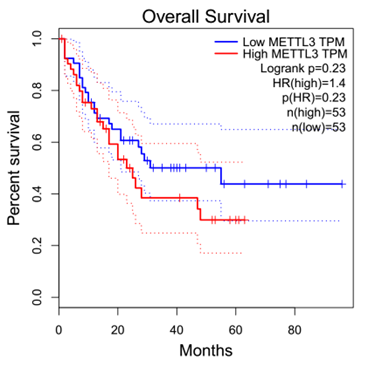** | **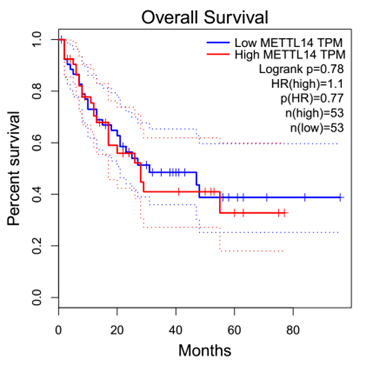** | **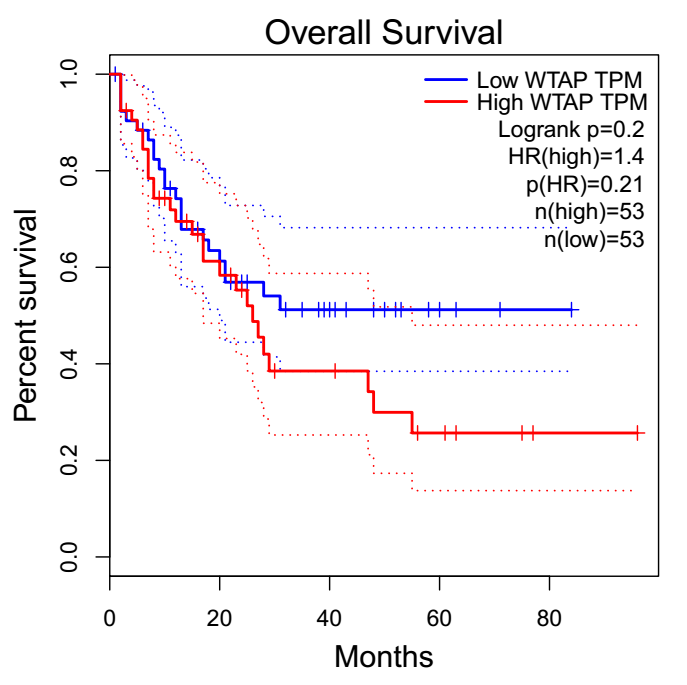** |
| --- | --- | --- |
| **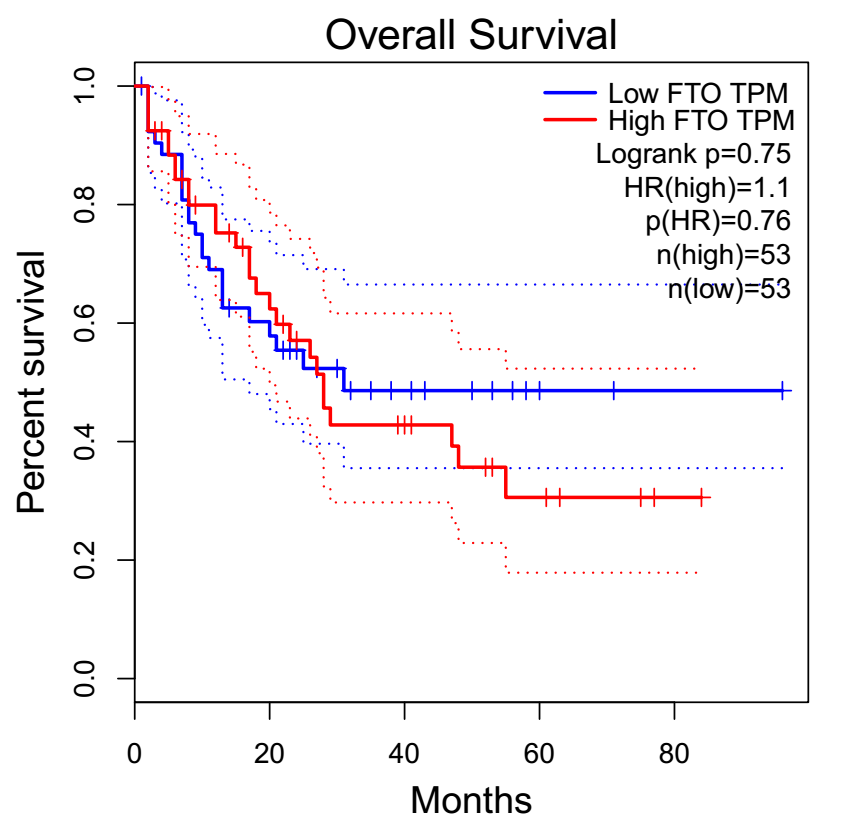** | **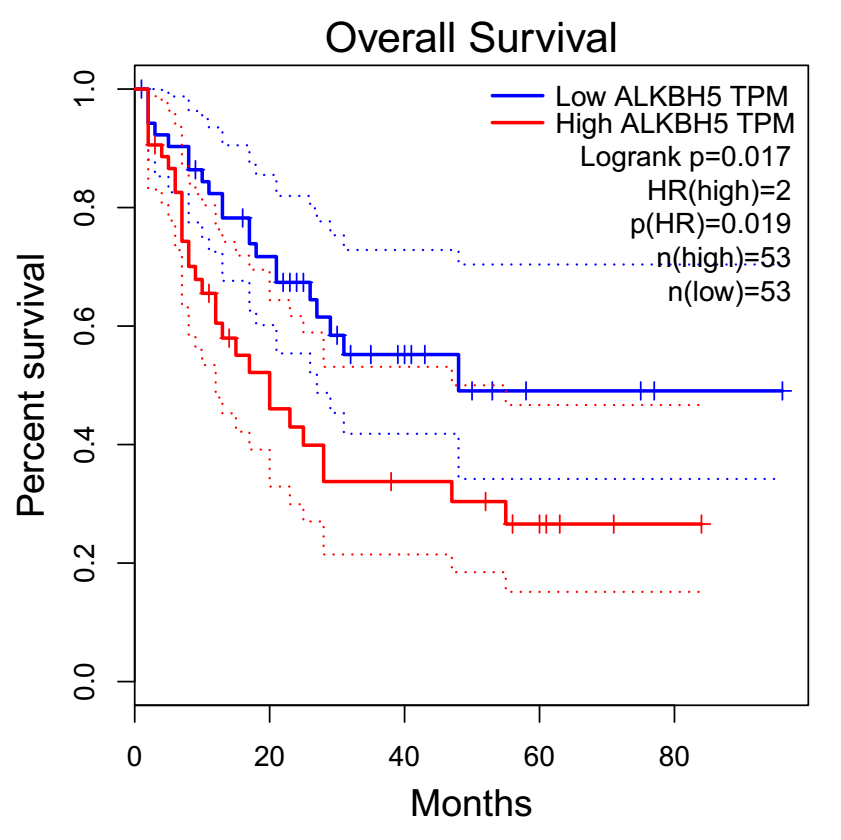** |  |
| **Supplement figure 4** Overall Survival (OS) of low and high expression levels of METTL3, METTL14, WTAP, FTO, and ALKBH5 (n=206) from TCGA dataset | | |

| 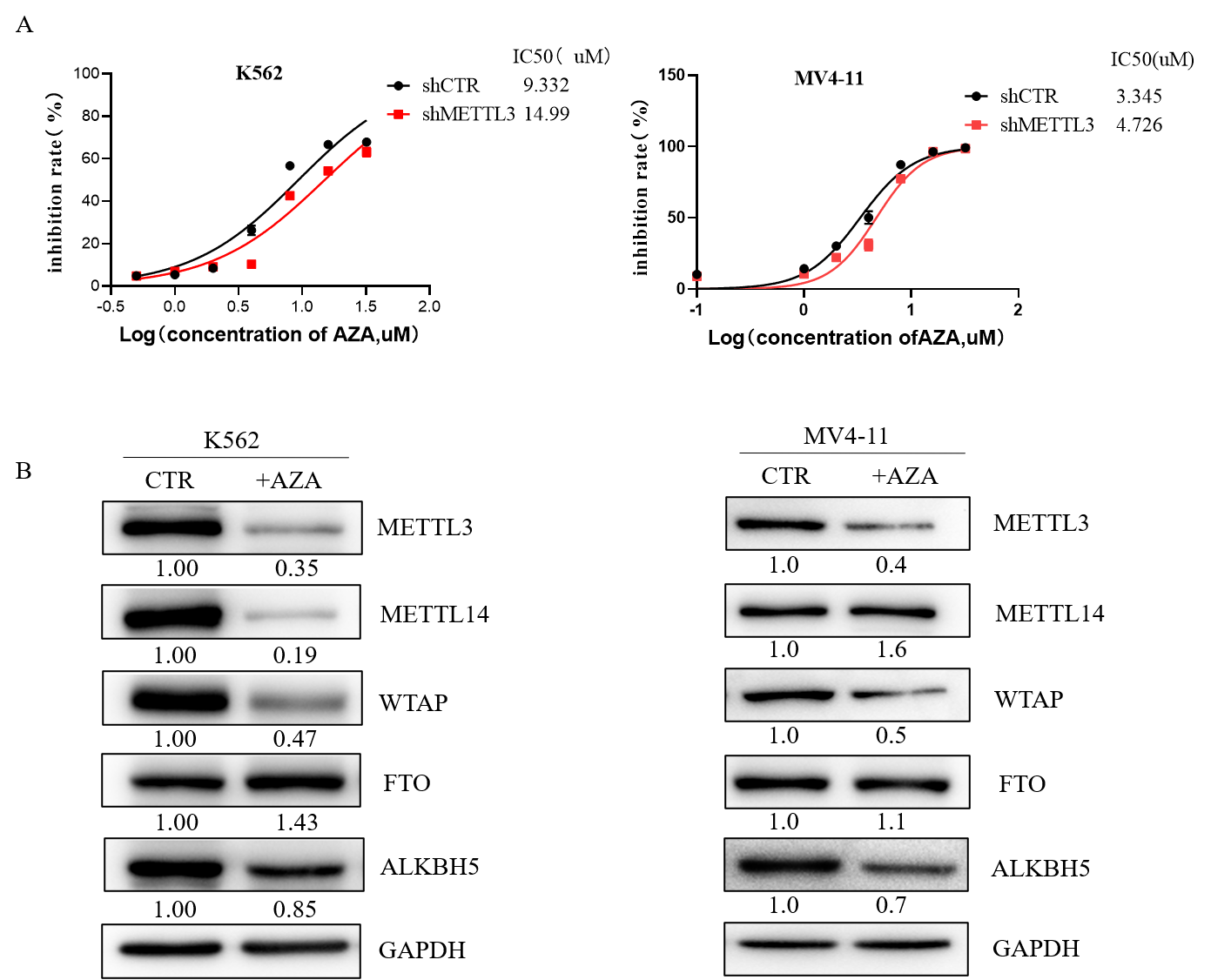 |
| --- |
| **Supplement figure 5** IC_50_ of azacytidine in AML cells with METTL3 knockdown |
|  |
| **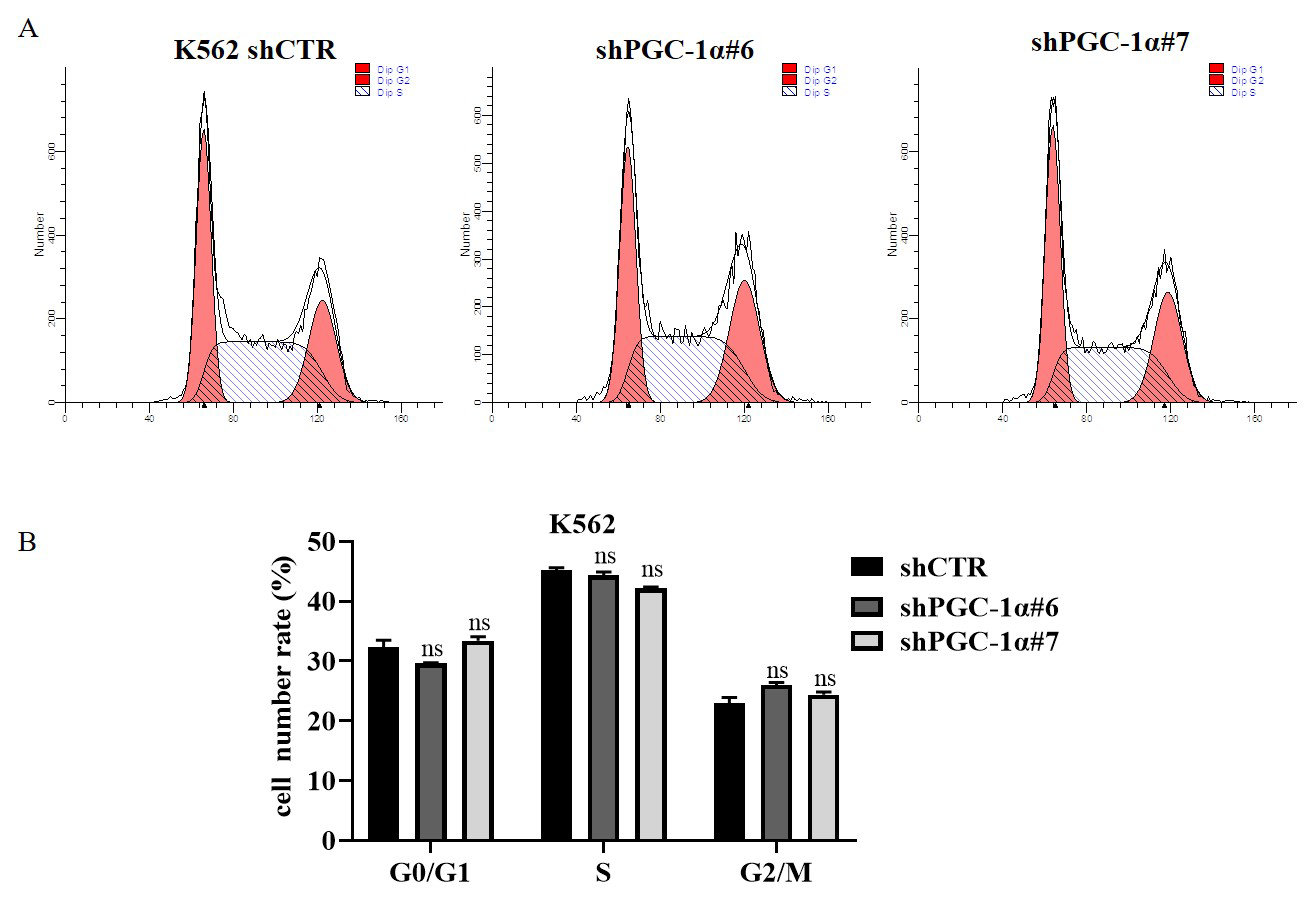** |
| **Supplement figure 6 Effect of PGC-1α on cell cycle in AML cells** (A) The cell cycles of K562 shCTR and K562 shPGC-1α#6 and K562 shPGC-1α#7 were detected by flow cytometry. (B) The result A is represented in a bar chart, and the result is the mean ± SD of 3 replicates. vs CTR: ns means p > 0.05. |
| **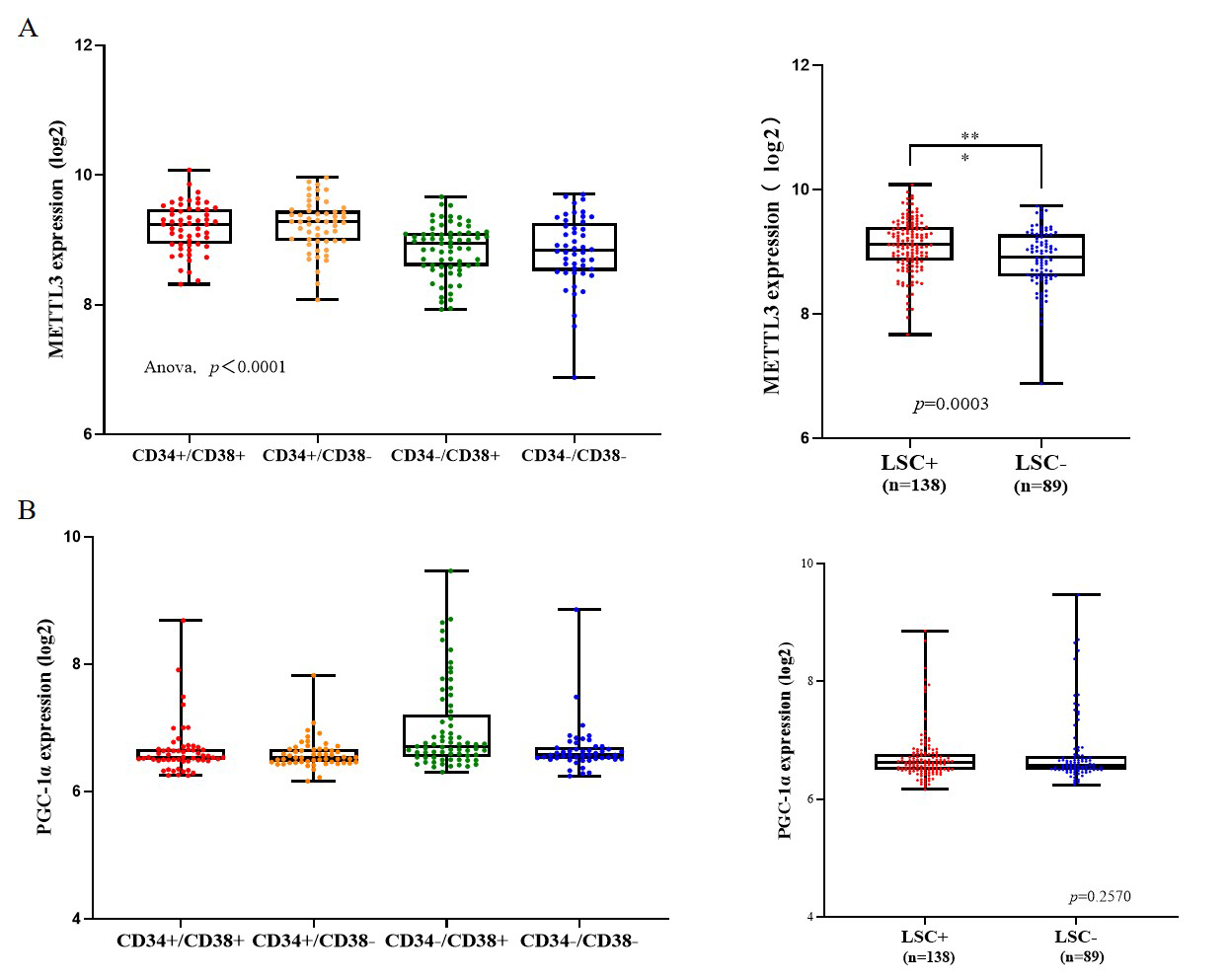** |
| **Supplement figure 7** Expression of METTL3 and PGC-1α in LSCs (GSE76009) |

| **Supplement Table 4** Comparison of clinical features of AML patients with METTL3-positive and METTL3-negative | | | |
| --- | --- | --- | --- |
| clinical features | METTL3+（n=90） | METTL3-（n=22） | P value |
| Gender, male/female | 43/47 | 11/11 | 0.852 |
| Age (years), median age (range) | 46（14-84） | 47（21-73） | 0.836 |
| FAB classification |  |  |  |
| M0, % (cases/total) | 1.1（1/90） | 0%（0/22） | 1.000 |
| M1, % (cases/total) | 27.8（25/90） | 4.5（1/22） | 0.021 |
| M2, % (cases/total) | 25.6（23/90） | 40.9（9/22） | 0.153 |
| M3, % (cases/total) | 7.8（7/90） | 40.9（9/22） | 0.000 |
| M3, % (cases/total) | 26.7（24/90） | 9.1（2/22） | 0.080 |
| M3, % (cases/total) | 11.1（10/90） | 4.5（1/22） | 0.689 |
| Baseline parameters at first visit |  |  |  |
| WBC (×10^9^/L), mean ± SD | 65.63±86.34 | 33.70±50.69 | 0.028 |
| HB (g/L), mean ± SD | 85±24 | 82±19 | 0.589 |
| PLT(×10^9^/L), mean ± SD | 64±93 | 59±82 | 0.806 |
| Blasts in peripheral blood (%), mean ± SD | 51.3±35.3 | 37.8±38.1 | 0.142 |
| Blasts in bone marrow, mean ± SD | 65.7±25.2 | 60.3±31.6 | 0.509 |
| LDH level, elevated/normal | 80.0（72/90） | 81.8（18/22） | 0.847 |
| Prognostic stratification |  |  |  |
| Low risk group, % (cases/total) | 32.1（25/78） | 57.1（12/21） | 0.035 |
| Intermediate risk group, % (cases/total) | 39.7（31/78） | 28.6（6/21） | 0.348 |
| High-risk group, % (number of cases/total) | 28.2（22/78） | 14.3（3/21） | 0.193 |
| Gene mutation |  |  |  |
| CEBPA double +, % (cases/total) | 15.1（11/73） | 5.6（1/18） | 0.448 |
| NPM1+, % (cases/total) | 21.9（16/73） | 11.1（2/18） | 0.509 |
| FLT3-ITD+, % (cases/total) | 28.4（21/73） | 16.7（3/18） | 0.381 |
| DNMT3A+, % (cases/total) | 20.5（15/73） | 0（0/18） | 0.036 |
| KIT+, % (cases/total) | 6.8（5/73） | 11.1（2/18） | 0.543 |
| TP53+, % (cases/total) | 3.0（2/67） | 7.1（1/14） | 0.454 |
| ASXL1+, % (cases/total) | 6.0（4/67） | 28.6（4/14） | 0.010 |
| IDH2+, % (cases/total) | 13.4（9/67） | 21.4（3/14） | 0.444 |
| IDH1+, % (cases/total) | 9.0（6/67） | 7.1（1/14） | 0.826 |
| TET2+, % (cases/total) | 9.0（6/67） | 0（0/14） | 0.245 |
| RUNX1, % (cases/total) | 7.5（5/67） | 7.1（1/14） | 0.967 |
| Extramedullary infiltration |  |  |  |
| Hepatosplenomegaly, % (cases/total) | 13.3（12/90） | 0%（0/22） | 0.070 |
| Lymphadenectasis, % (cases/total) | 25.6（23/90） | 9.1（2/22） | 0.096 |
| Central nervous system leukemia, % (cases/total) | 4.4（4/90） | 4.5（1/22） | 0.984 |
| Therapeutic response |  |  |  |
| 1 course response rate, % (cases/total) | 62.0（44/71） | 77.8（14/18） | 0.209 |
| 2 course response rate, % (cases/total) | 80.3（57/71） | 100（18/18） | 0.064 |
| Recurrence rate, % (cases/total) | 32.1（25/60） | 44.4（8/18） | 0.318 |
